# Supplementary figures and images for: Functional dynamics between resident transcriptionally active microbes (TAMs) and host genes underlie Dengue severity
Source: PLoS Negl Trop Dis. 2025 Dec 19;19(12):e0013836. doi: 10.1371/journal.pntd.0013836 (PMC12716792; doi:10.1371/journal.pntd.0013836)

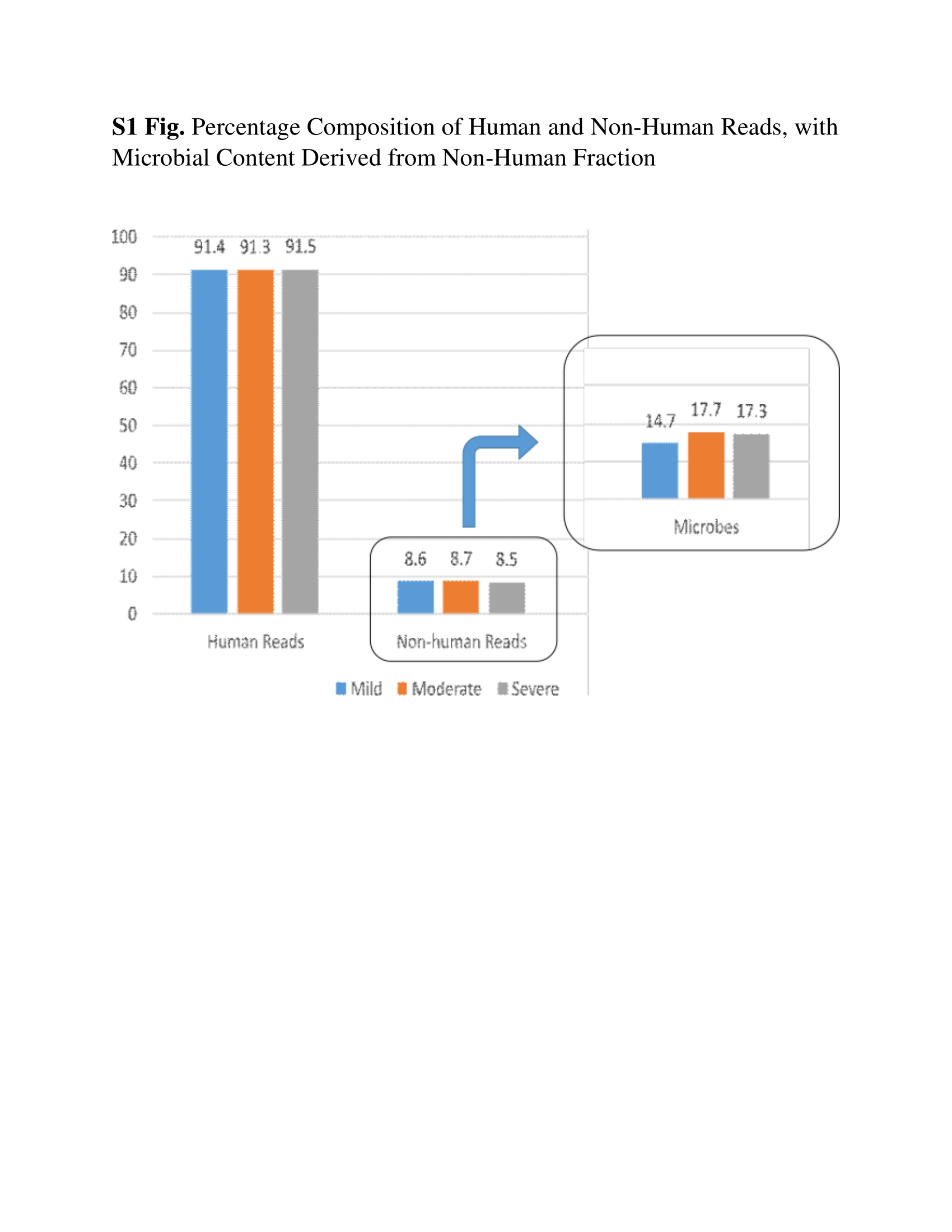

Supplement: S1 Fig — (TIFF) [file pntd.0013836.s001.tiff]

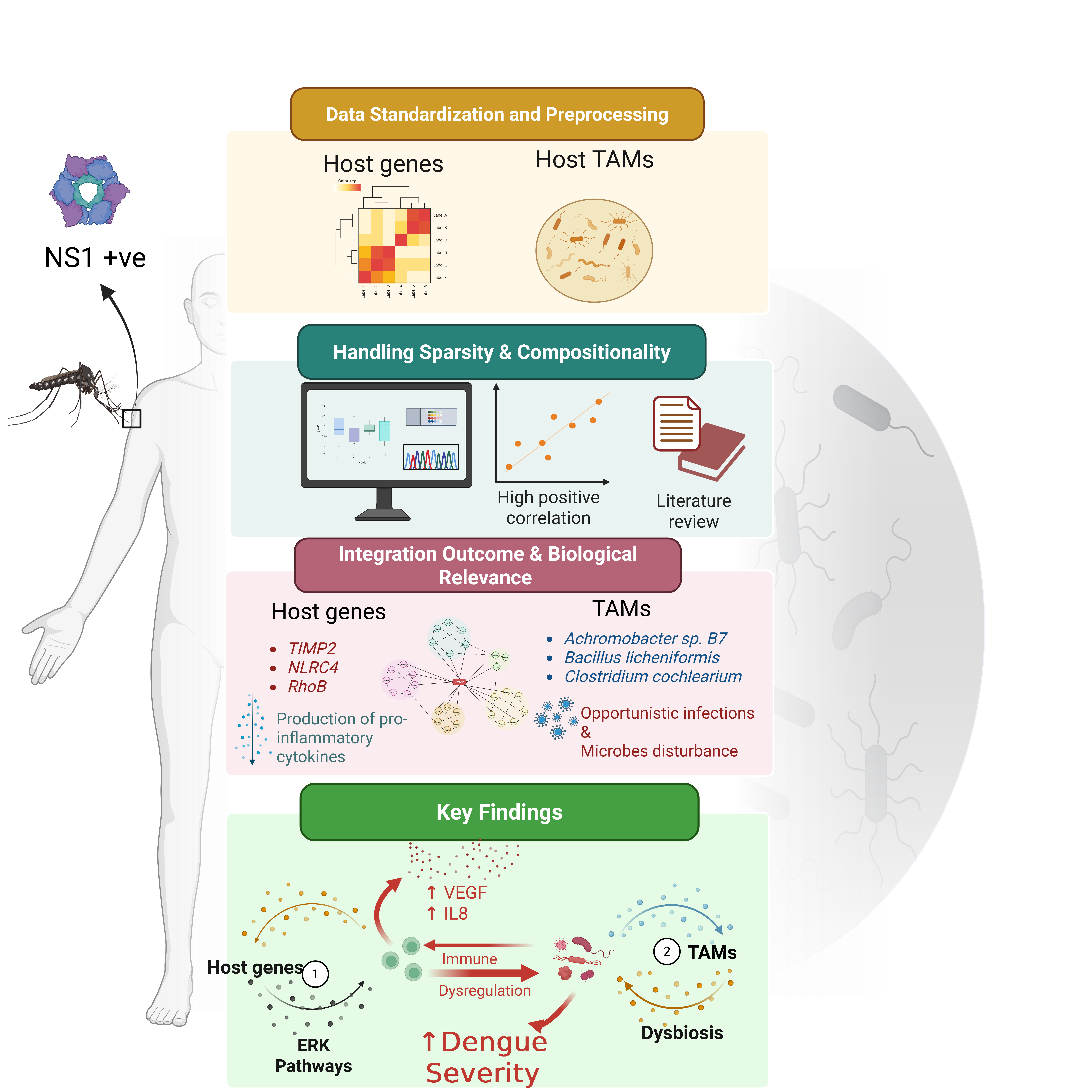

Supplement: S2 Fig — (Figure created with Biorender.com. Devi, P. (2025) https://BioRender.com/neii33a). (TIFF) [file pntd.0013836.s002.tiff]
