# Supplementary material for: Functional dynamics between resident transcriptionally active microbes (TAMs) and host genes underlie Dengue severity
Source: PLoS Negl Trop Dis. 2025 Dec 19;19(12):e0013836. doi: 10.1371/journal.pntd.0013836 (PMC12716792; doi:10.1371/journal.pntd.0013836)
Supplement: Attachment — Submitted filename: Response to Reviewers.docx [file pntd.0013836.s012.docx]

We thank the Editor for facilitating the peer-review of the submitted manuscript. We also take this opportunity to acknowledge and appreciate the anonymous reviewers for doing the needful. We have addressed all the suggestions/queries from the reviewers and details for which are captured in the ensuing response to reviewers.

Best wishes,

Rajesh

The Methods section requires substantial improvement because it is missing several key details, including information on pathway analysis, enrichment, gene expression analysis, etc.

We extend our gratitude to the reviewer’s kind evaluation of methodology section in detail. In the revised submission, we have updated the information regarding the pathway analysis in the main manuscript and incorporated it as mentioned below.

“**Functional Pathway Enrichment Analysis:**

Functional pathway of human mapped genes extracted from DESeq2 analysis was performed using the Enrichr web tool (https://maayanlab.cloud/) utilizing pathway and term annotations from the Reactome database. Pathways with an adjusted p-value ≤ 0.05 were considered statistically significant. Second filter was applied on the basis of the genes present in the particular pathway, for ex: pathways containing fewer than 25 or more than 300 genes were excluded to avoid biases in the pathway. This filtering ensured the retention of pathways with biological relevance. The results were visualized in R using the ggplot2 package, where the combined score was plotted against the number of genes associated with each pathway. In the plots, adjusted p-values were represented by color gradients, and bubble size indicated gene counts present in the particular pathway.”

The human reference assembly is a specific haplotype, and not all sequences will map to it. For validation, BLAST should be performed on the unmapped data. Did the authors also try the Telomere-to-Telomere (T2T), the first complete, gapless sequence of a human genome?

We thank the reviewer and acknowledge that the GRCh38 reference genome represents a single haplotype and may not capture all the genomic variation, which may result in a fraction of reads remaining unmapped. Being aware of this possibility and to account for that, we performed offline BLAST+ on the total unmapped reads and observed hits with human origin. This may be due to the differences in alignment stringency between HISAT2 and BLAST - while HISAT2 uses short-seed alignment with mismatch penalties, BLAST performs local alignments and can detect partial similarities. The possible reasons for the presence of human-origin reads among the unmapped reads include (https://daehwankimlab.github.io/hisat2/manual/):

- If one read of a pair maps successfully and the other fails to align, HISAT2 includes the unmapped mate in the “unmapped” file.
- Variations such as SNPs or small insertions/deletions may prevent exact matches with the reference genome, causing HISAT2 to miss these reads.
- Reads with excessive ambiguous bases (Ns) may fail to align properly.

These factors collectively contribute to the presence of human-origin sequences within the unmapped reads. Even after the above stringency, in our study, around 20% of the unmapped human reads were identified as microbial in origin, indicating that the unmapped human reads need closer attention. Further to confirm the authenticity of these microbial reads, we re-mapped these microbial classified reads back to the human reference genome (GRCh38). For that analysis, the reads classified via kraken2 were extracted using the **“extract_kraken_reads.py”** script from the KrakenTool package. The resulting FASTQ files were converted to FASTA format using **Seqtk**, and the reads were then concatenated using **SPAdes**. Subsequently, an offline **BLAST+** search was performed to map these reads against the human reference genome, and the BLAST output returned zero hits. These findings verify that the mapped sequences represent microbial reads alone, confirming the absence of human reads.

We used GRCh38 human reference in our study, as it is the current standard and widely adopted reference for human transcriptomic studies. GRCh38 provides comprehensive gene annotations and is fully supported by major databases such as Ensembl, GENCODE, and RefSeq, ensuring compatibility with commonly used RNA-seq and downstream analysis tools. While the Telomere-to-Telomere (T2T-CHM13) assembly offers a more complete genome, its annotations and tool integration are still under development, and our primary objective was to identify transcriptionally active microbial reads rather than the host genomic features. Therefore, GRCh38 was selected to maintain reproducibility, compatibility, and consistency with established analytical workflows.

Although the database tested for taxonomy classification includes viruses, it's surprising that viral reads have not been reported. Were any dengue virus reads identified?

We thank the reviewer for carefully reviewing the work and for prompting us to further improve the clarity of our findings. Yes, the database used for taxonomy classification includes viral sequences along with bacterial and archaeal databases and we did identify viral reads, including dengue virus, as well as the archaeal reads. Some of the archaeal taxa have already been mentioned in the final results in **supplementary file 6 (S6 file)**. In the revised submission, we have included average count of dengue virus (table below) and an excel file with this revision of all the microbial species, including the detected viral and archaeal reads, as new **supplementary file 7 (S7 file)**.

| Dengue (Biotype) | Average count |
| --- | --- |
| Mild | 22005 |
| Moderate | 4473 |
| Severe | 5006 |

kraken2 DB name and version missing.

We thank the reviewer for highlighting this. We would like to clarify that the database is downloaded from Minikraken v1. It contains Refseq: bacteria, archaea, viral, Index size (8GB), and *kraken2-build* was used to build the database (<https://benlangmead.github.io/aws-indexes/k2>). The details have now been included in the revised submission in the methodology section (line 506-507).

Details needed for QC and filtering of meta-transcriptomic reads.

We extend our gratitude to the reviewer for this insightful suggestion. We would like to highlight that for the QC of meta-transcriptomic reads, the microbial classified reads were mapped back with the human reference genome (GRCh38) to ensure removal of any potential human origin sequences. For that analysis, the reads classified via Kraken2 were extracted using the **“extract_kraken_reads.py”** script from the KrakenTool package. The resulting FASTQ files were converted to FASTA format using **Seqtk**, and the reads were then concatenated using **SPAdes**. Subsequently, an offline **BLAST+** search was performed to map these reads against the human reference genome, and the BLAST output returned zero hits. The source code for the analysis described here is publicly available at <https://github.com/pallawikumari/-Metagenome>. These findings verify that the mapped sequences represent microbial reads alone, confirming the absence of human reads. This screenshot indicates the result, which shows zero hits.

Further, to filter mapped and unmapped reads, the BAM files were processed using Samtools with the following commands:

$ samtools view -b -f 2 filename.bam > Mapped.bam

This command extracts only the properly paired reads from the BAM file, retaining reads that are correctly aligned as pairs.

$ samtools view -b -F 2 filename.bam > Unmapped.bam

This command removes properly paired reads, keeping only unpaired or discordant reads, which can then be used for further analysis of unmapped sequences.

Is the normalization of the relative abundance obtained from Bracken relevant? This normalization may have been performed prior to the taxonomic or relative abundance analysis.

We acknowledge the reviewer’s concern regarding the normalization of the relative abundance obtained from Bracken2. We are pleased to clarify that initially, Kraken2 was used for taxonomic classification of the non-human reads, providing a preliminary mapping of the reads to microbial taxa. The output from Kraken2 was then processed using Bracken2, which refines the taxonomic classification. In our study, we used raw counts from Bracken2 and applied Cumulative Sum Scaling (CSS) transformation to normalize before calculating relative abundances. This normalization approach adjusts for differences in library sizes across samples by scaling each sample’s counts based on a cumulative sum, thereby minimizing biases in relative abundance comparisons caused by variations in sequencing depth. This approach aligns with best practice in microbiome and meta-transcriptomic studies, ensuring reliable interpretation of taxonomic abundance patterns.

Despite its strengths, Lasso regression has some limitations. How do the authors address the bias caused by regularization and the issue of highly correlated features? Instead of distributing weights among correlated features, Lasso may arbitrarily select one and ignore the others, potentially missing useful information.

We acknowledge the reviewer perspective. Our rationale for LASSO includes that this method has demonstrated success across a wide range of applications, although we acknowledge that it also presents several limitations, as outlined below:

(n=number of samples, p=number of features) *

(a) In situations where p > n, LASSO can select at most n variables before reaching saturation due to the constraints of the convex optimization problem. This characteristic limits its effectiveness as a variable selection method in high-dimensional settings. Furthermore, the LASSO solution is not well-defined unless the bound on the L1-norm of the coefficients remains below a certain threshold.

(b) When a group of predictors exhibits strong pairwise correlations, LASSO typically selects only one variable from the correlated set, without preference for which specific variable is chosen. This can lead to instability and loss of potentially informative predictors.

(c) In cases where n > p, empirical evidence suggests that when predictors are highly correlated, the predictive performance of LASSO is often surpassed by that of ridge regression (Tibshirani, 1996).

Being aware of the above possibilities, we were stringent in our study, wherein we applied LASSO-penalized regression to identify specific associations between individual host genes and microbial taxa within each dengue cohort (Mild, Moderate, and Severe). For this analysis, a modeling approach was employed, in which the abundances of microbial taxa were treated as the response variable, and the expression level of each host gene served as predictor. LASSO regression incorporates an L1 regularization penalty to perform both shrinkage and variable selection, thereby identifying only the most relevant taxa correlated with each host gene’s expression. This approach not only reduces the bias introduced by the regularization but also enables the detection of relevant associations across all the taxonomic levels (PMID: 37443060). Moreover, it provides an efficient solution for analyzing large, high-dimensional datasets such as microbiome and host gene expression data, while maintaining computational feasibility and biological interpretability (Regression Shrinkage and Selection via the Lasso, https://doi.org/10.1111/j.1467-9868.2010.00740.x).

In addition to applying stability selection, we further refined our results through an extensive literature review, selecting only those host genes and microbial taxa that demonstrated biologically relevant functions in the context of the disease. We have included the conceptual image to provide a clear and comprehensive visual summary of the analyses performed in this study **(Figure 5)**.

Abbreviations should be detailed the first time they are mentioned in the text.

In the revised submission, we have ensured that all abbreviations are defined in full upon their first appearance in the text.

Very low levels of microbial reads mapped to the Kraken2 database. This clearly highlights the low level of mapping.

We agree with the reviewer's observation; however, the low microbial read proportion in our study is consistent with expectations from human blood-derived RNA-seq datasets, where microbial biomass is minimal and host RNA predominates. In the holo-transcriptomic/meta-transcriptomic based approaches, the percentage of RNA-seq reads mapping to microbes would be lower compared to the human host. At the same time, it is dependent on the sampling site, which is dependent on the disease being investigated. For e.g., studies from our lab during COVID-19 have shown that around ~30-35% of the RNA-seq reads are non-human reads. This is because nasopharyngeal RNA was used for the COVID-19 studies. This is different for Dengue, where venous blood is used for study in alignment with the dengue disease pathophysiology. There are comparable findings reported in other studies analyzing blood-derived transcriptomes. For example, Simon et al. (2018) reported that 90.7% of RNA-seq reads mapped to the human genome, with only 0.20% assigned to bacterial metafeatures (https://doi.org/10.1093/gigascience/giy070). Similarly, Sirasani et al. (2025) noted that low microbial abundance in blood and many tissue types results in host RNA overwhelmingly dominating sequencing reads (https://doi.org/10.1093/bib/bbaf176). This also underscores the sensitivity of our approach, which was still able to detect and classify a subset of transcriptionally active microbial reads despite the host-dominated nature of the sample.

The read numbers don't correspond to those present in the S1 file.

We thank the reviewer. The read numbers have now been verified and updated to ensure consistency with the information provided in **Supplementary File S1**.

The PCoA graph is too small to follow, but there does not seem to be any clustering pattern. The Shannon and Simpson indices of the sample are very similar, highlighting that there is no significant difference within the samples.

In acknowledgement of the reviewer suggestion, the beta diversity (PCoA) plot has been updated in the revised manuscript to improve clarity and readability. As correctly noted, the alpha diversity analysis (Shannon and Simpson indices) shows no significant difference among the samples. We are including the image Shannon and Simpson indices with p-value:

Figure 2 has way too many subgraphs, and they are too small with small text, making it difficult to follow up on.

We thank the reviewer for this valuable observation. The figure has been revised in the updated manuscript - the layout and font size have been improved to enhance readability and clarity.

**Figure 2:** **A)** Box plot reflects the alpha diversity, specifically, **I)** Shannon, **II)** Simpson, which is measuring within sample species richness and evenness, respectively, and **III)** Beta diversity compares microbial composition between samples, highlighting community dissimilarity. It is visualized using Principal Coordinates Analysis (PCoA) based on the distance matrices like Bray-Curtis. **B)** The total number of distinct phylum identified within each severity group (mild, moderate, and severe) reflects in the **B(I)**, **(II)** represents the number of genera present in each group, offering a more detailed view of the microbial composition changes. Total number of TAMs, genes and their distribution throughout the group is shown in (III) & (IV). **C)** The Procrustes analysis visualizes the concordance between microbial and gene expression datasets by superimposing their ordination plots across I) Mild, II) Moderate, and III) Severe.

It’s incorrect to say that there is a non-human fraction. As the mapping shows, < 20% of the unmapped reads mapped to the microbial DB.

We agree that the proportion of unmapped reads aligning to the microbial database is <20%. We have updated the word non-human to human-unmapped reads in the revised submission.

Why is the study considered multi-omics when only the transcriptomics dataset has been analyzed?

The study is considered multi-omics because it integrates both **host (human)** and **microbial** transcriptomic data derived from the same hospital-admitted patients. While both datasets are transcriptomic in nature, they represent two distinct biological domains: the host and its associated microbiome, and their combined analysis provides a multi-omics perspective on host-pathogen interactions during dengue infection.

However, we fully acknowledge the reviewer’s point and are therefore open to revising the terminology in the manuscript to more precisely describe the study as an **integrated host-microbial transcriptomic analysis** rather than a multi-omics study.

Figure 5 is unnecessary; it's more suited for a presentation or poster rather than a scientific publication.

We included this conceptual image to provide a clear and comprehensive visual summary of the study findings. The intention was to augment a wider readership for non-directly aligned readers to have take-home message. We would request for it being there in the manuscript for augmenting readership.

Without line numbers, it is difficult to provide specific comments for the corresponding lines.

We have now included line numbers in our revised submission.

No Dengue virus reads have been reported. Although the Krakten2 DB did include a virus. Could the author comment on this?

We have responded to this query above wherein we have included the Dengue virus reads in the revised submission.

It is interesting to see that several microbial reads have been reported. Human blood is traditionally considered sterile; however, recent studies have raised questions about the existence of a blood microbiome in healthy individuals.

A recent study from Tan et al. (<https://www.nature.com/articles/s41564-023-01350-w>) suggests that there does not exist a consistent core microbiome endogenous to human blood—instead, a transient and sporadic translocation of commensal microbes from other body sites into the bloodstream. No species were detected in 84% of individuals (n = 9770), while the remainder had a median of only one species.

We agree that the concept of a blood microbiome remains a topic of active debate (https://doi.org/10.3389/fcimb.2019.00148). Traditionally, blood has been considered a sterile environment; however, several recent studies have highlighted alternative findings by detecting microbial DNA, RNA, or metabolites in the blood from both healthy and diseased individuals. Existing evidence also indicates that blood microbiome dysbiosis may be associated with disease progression and host immune modulation in diseases such as cardiovascular disease, liver cirrhosis, pancreatitis, type 2 diabetes, cancer, and COVID-19.

While a few reports, as mentioned by reviewer Tan et. al, find no evidence for a consistent core blood microbiome, instead supporting the hypothesis of transient microbial translocation from other body sites such as the gut, oral cavity, and skin. Such translocation can occur through mucosal breaches or loose intestinal permeability. We were aware of the Tan et al. study and the findings therein, but we noted that it is a multi-cohort study of healthy humans over a period of time. It is important to note that we have investigated only the confirmed Dengue virus infected hospitalized patients with differential disease severity based on the clinical data (mild, moderate, severe) and not healthy humans. In such pathological conditions, barrier dysfunction and systemic inflammation can facilitate transient microbial translocation into the bloodstream, which are transcriptionally active and interacts with the host immune system. Accordingly, our findings suggest that microbial components may circulate and potentially modulate host responses, contributing to the variations in disease severity. Being aware of the importance, we have been stringent vis-à-vis the bioinformatics pipeline implemented in the study. Based on the previous work from the lab in this domain, in addition to the research articles on this area of research, we have published a STAR Protocol which captures all the details vis-à-vis the bioinformatics pipeline for the transcriptionally active microbes’ (TAMs) detection and elucidating their potential role in disease severity, (<https://doi.org/10.1016/j.xpro.2024.103071>, *Protocol to decode the role of transcriptionally active microbes in SARS-CoV-2-positive patients using an RNA-seq-based approach*).

In the context of dengue, our findings similarly support the presence of active microbial components that may circulate transiently and modulate host responses, potentially influencing clinical outcomes.
