# Supplementary material for: Functional dynamics between resident transcriptionally active microbes (TAMs) and host genes underlie Dengue severity
Source: PLoS Negl Trop Dis. 2025 Dec 19;19(12):e0013836. doi: 10.1371/journal.pntd.0013836 (PMC12716792; doi:10.1371/journal.pntd.0013836)
Supplement: Attachment — Submitted filename: Response to Reviewer 2_PNTD-D-25-01147R1.docx [file pntd.0013836.s013.docx]

We thank the Editor for facilitating the peer-review of the submitted manuscript. We also take this opportunity to acknowledge and appreciate the anonymous reviewers for their time and effort towards the peer-review. We have incorporated suggestions from the reviewers and details for which are captured in the ensuing response to reviewers.

Best wishes,

Rajesh

- Some multi-panel visuals, especially the one on species variety and classification, are still packed tightly, making text hard to read. Bigger fonts plus larger sections could help clarity a lot.

We thank the reviewer for this helpful comment. In response to this, we have updated the corresponding multi-panel figure by increasing the font size and enlarging the sections displaying species name.

**Fig 4: Machine learning model identifies biological pathways affected in different disease severities. A)** Canonical steps for interpreting and selecting pathways and their associated genes, representing the Lasso regression model to uncover potential microbial contributors linked to these genes. **B)** Mild, and **C)** Severe: **I)** Pathways selected on the basis of biological relevance are shown though bubble plot, **II)** Network represent the selected genes and their associated pathways with TAMs based on significance level. Figure created with licensed version of Biorender.com.

- A few abbreviations like TLC or SGOT are used before explaining what they mean. Since AST is also introduced without definition, spelling out each one early would help a wider audience grasp them faster.

We thank the reviewer for pointing this out. We have now revised the manuscript to ensure that all abbreviations, including TLC, SGOT, and AST, are defined at their first appearance to improve clarity for a broader audience.

- Figure 5 can be moved to the supplementary information.

We thank the reviewer for this suggestion and now we have put Figure 5 as a supplementary image (S2 Fig).

**S2 Fig.** Illustrates significant host gene-TAMs associations and their functional roles in modulating immune and inflammatory pathways contributing to severe dengue. Figure created with Biorender.com

- Thank you for updating the Dengue virus read counts. However, viral read counts are 4-5 times higher in mild patients than in those with moderate or severe cases. Could they discuss in the paper why this discrepancy exists?

Thank you for the observation regarding the higher viral read counts in mild dengue cases (Mild: 22005, Moderate: 4473, Severe: 5006).

“As we have taken samples on the first day when symptomatic patients reported to the hospital for testing, we are unable to pinpoint the exact day on infection. Thus different viral counts between mild and moderate/severe may be reflective of the different time from infection to their reporting to the hospital for testing. At the same time, clinical parameters affirm the differential disease severity.”

We have now discussed this in the first result of the revised submission (line no. 135-141).

- Please modify the language and remove multi-omics.

We thank the reviewer for accepting our request and now we are changing the phrase as **integrated host-microbial transcriptomic analysis** instead of multi-omics study.

- Please mention and discuss the blood microbiota, especially the study by Tan et al., which has a huge cohort of nearly 10,000.

We thank the reviewer for their suggestion. In response, we have now incorporated the findings from Tan et al. into the discussion (395-401):

"While studies such as Tan et al. report no evidence of a consistent core blood microbiome in a large multi-cohort meta-analysis of 9,770 healthy individuals, it is important to emphasize that our study focuses specifically on the confirmed Dengue virus–infected hospitalized patients with varying disease severity (mild, moderate, severe), rather than healthy individuals. In pathological conditions such as Dengue infection, barrier dysfunction and systemic inflammation can impact transient microbial translocation into the bloodstream. These translocated microbes may remain transcriptionally active and interact with the host immune system, thereby contributing to disease progression."
